# Metastatic Melanoma Causing Superior Vena Cava (SVC) Syndrome: A Case Report of a 65-Year-Old Male With a History of Multiple Myeloma and Melanoma

**DOI:** 10.7759/cureus.63522

**Published:** 2024-06-30

**Authors:** Mohammad Q Jafri, Ahmad Abu Homoud

**Affiliations:** 1 Internal Medicine, Ocean University Medical Center, Brick, USA; 2 Pulmonary and Critical Care Medicine, Jersey Shore University Medical Center, Neptune, USA

**Keywords:** malignancy-related superior-vena-cava-syndrome, endobronchial ultrasound (ebus), mediastinal masses, superior vena cava (svc) syndrome, malignant melanoma metastasis

## Abstract

A 65-year-old male with a history of multiple myeloma and melanoma presented to the hospital with shortness of breath and lightheadedness. He was subsequently diagnosed with mild superior vena cava (SVC) syndrome due to a metastatic melanoma mediastinal mass. While melanoma frequently metastasizes to the lungs, the occurrence of SVC syndrome resulting from metastatic melanoma is exceedingly rare compared to other malignancies like lung cancer. Consequently, data on the incidence or prevalence of SVC syndrome caused by metastatic melanoma are sparse and variable. This case particularly underscores the rarity of melanoma causing SVC syndrome, as evidenced by the oncology team's request to perform a second biopsy to confirm the diagnosis. This case also highlights the need for a tailored diagnostic and management approach, providing valuable insights into the diverse presentations of melanoma and enriching the medical literature on this subject.

## Introduction

Superior vena cava (SVC) syndrome occurs when blood flow through the SVC is partially or fully blocked, usually due to a blood clot or tumor growth. The SVC is a major vein that returns blood from the upper body to the heart. This syndrome is often caused by cancer but can also result from non-cancerous conditions, leading to symptoms like swelling in the face, neck, and arms; difficulty breathing; coughing; and enlarged chest veins [1]. Approximately 15,000 cases of SVC syndrome are noted annually in the United States, with incidence rates ranging from one in 650 to one in 3100 patients [1]. Most cases of SVC syndrome are due to cancers in the mediastinum, particularly small-cell bronchogenic carcinoma. Lung cancer is the most common cause of SVC syndrome, responsible for about 60%-85% of cases [2]. Both small-cell lung cancer (SCLC) and non-small-cell lung cancer (NSCLC) can lead to SVC syndrome, with SCLC being more commonly associated due to its central location and aggressive nature. [2]. Lymphomas, particularly non-Hodgkin lymphoma, account for about 10%-20% of SVC syndrome cases [3].

Melanoma ranks as the third most common skin cancer, with incidences growing in recent years to become the fifth most common cancer overall by 2023 [4]. Metastatic melanoma (MM) had an incidence of 0.9 per 100,000 between 2014 and 2018, with pulmonary metastases being the primary cause of death in MM, leading to respiratory failure [4]. The estimated incidence of pulmonary MM of the lung accounts for 0.01% of all primary lung tumors [5]. Although melanoma has a propensity for metastasizing to the lungs, the occurrence of SVC syndrome due to MM is relatively uncommon compared to other malignancies like lung cancer. Therefore, specific data on the incidence or prevalence rate of SVC syndrome caused by MM are limited and may vary widely.

A CT scan with contrast is used to screen for lung involvement, with metastatic nodules typically found at the lung periphery, often well-defined and around 1-2 cm in size. Here, we present a case of MM leading to a mediastinal mass that is likely causing mild SVC syndrome.

## Case presentation

A 65-year-old male with a medical history of multiple myeloma (s/p stem cell transplant in 2018, in remission, and on maintenance Revlimid) and melanoma of the right chest wall (s/p wide excision in 2000, which did not require adjuvant therapy or radiation, but with two out of several lymph nodes in his right axilla positive for metastases) presented to the hospital for shortness of breath associated with lightheadedness. He had been experiencing worsening dyspnea over the past month, feeling as though there was "cold water stuck" in his chest. The symptoms progressed, with the patient noticing facial swelling and erythema upon awakening the morning before admission. While showering, he acutely felt short of breath and lightheaded, prompting an emergency department (ED) visit.

**Figure 1:**
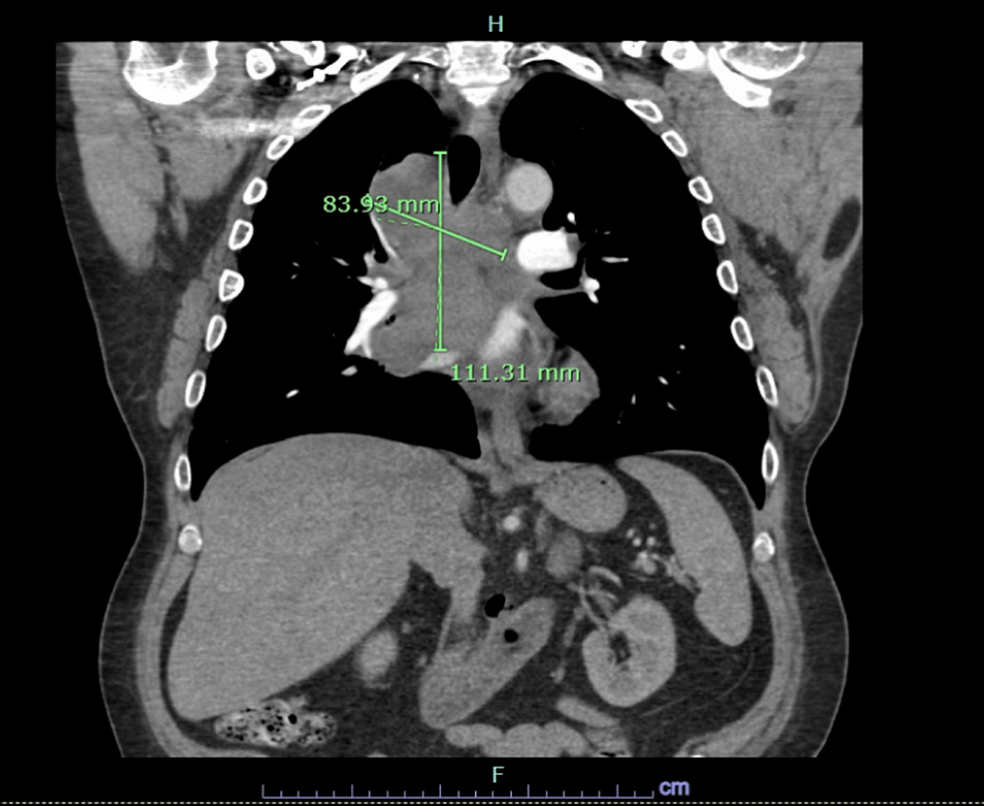
Coronal view of the large mediastinal mass with effect on the right pulmonary artery and the right middle and upper lobe airways measuring 111 x 84 cm

In the ED, the patient was slightly hypertensive and tachycardic but maintained good oxygen saturation on room air (Table 1). A CT chest with contrast revealed a large mediastinal mass with effect on the right pulmonary artery and the right middle and upper lobe airways (Figure 1). He was subsequently admitted to rule out malignancy and possible underlying SVC syndrome caused by the mediastinal mass. Empiric antibiotics and steroids were initiated. Of note, there was also no evidence of a new primary lesion in the skin at the time of this presentation.

**Table 1:** Initial set of vitals upon presentation along with height and weight

Vitals	Patient values
Temperature	97.6°F
Pulse	107 beats/min
Blood pressure	151/78 mmHg
Respiratory rate	19 breaths/min
Weight	95.3 kg (210 Ib)
Height	165.1 cm (5'5")

During his hospital course, his presenting symptoms eventually resolved, including his facial swelling and dyspnea, leading to the discontinuation of empiric antibiotics and steroids. He underwent a right-sided bronchoscopy with lavage and a right endobronchial ultrasound with biopsy (EBUS), which resulted in MM with small-cell features. Due to the rarity and unfamiliarity of MM causing SVC syndrome, oncology recommended that thoracic surgery be consulted for a mediastinoscopy with biopsy for confirmation of the diagnosis. Oncology also deemed that the patient was stable enough to follow up as an outpatient for the biopsy of the mediastinal mass.

**Figure 2:**
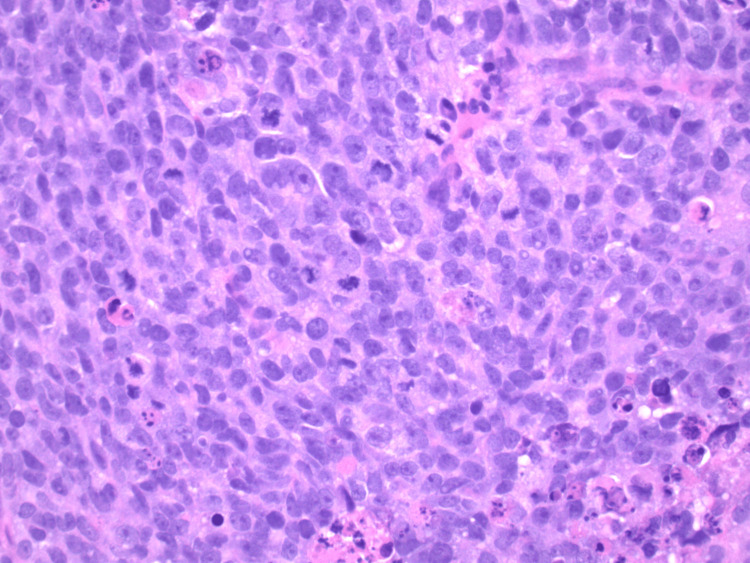
Poorly differentiated metastatic melanoma with small-cell features

About three weeks following this admission, the patient underwent a biopsy of the mass via mediastinoscopy, which confirmed poorly differentiated MM with small-cell features, specifically revealing morphologic features of highly necrotic tumor displaying fine chromatin and nuclear molding, as well as increased mitotic activity and apoptosis (Figure 2). At this point, the patient’s only complaint was dyspnea upon exertion, while he remained hemodynamically stable on room air. The hematology/oncology team planned to start the patient on nivolumab plus relatlimab as an outpatient.

## Discussion

Melanoma typically spreads locally and distantly, with metastasis risk linked to primary lesion depth. Early metastasis stages include invasion, angiogenesis, and colonization of target organs [4]. Even patients initially deemed node-negative can develop distant metastasis, and full lymph node removal has not shown clear survival benefits in node-positive cases [4]. Highlighting this point, the patient reported that two out of several lymph nodes in his right axilla were positive for metastases and were subsequently removed around the time of diagnosis about 24 years ago. Yet, the melanoma had still metastasized into this large mediastinal mass, very likely causing mild SVC syndrome.

As mentioned earlier, the occurrence of SVC syndrome due to MM is relatively uncommon compared to other malignancies like lung cancer. Primary malignant melanoma presenting as an anterior mediastinal mass without evidence of extra-thoracic disease is exceptionally rare, and melanoma primarily in the mediastinum has been documented in prior cases [6]. In one instance, it presented clinically as SVC syndrome, and the diagnosis was confirmed postmortem as primary malignant melanoma of the mediastinum. Another case involved amelanotic melanoma causing SVC syndrome [6].

In terms of treatment options for SVC, immediate radiation therapy (RT) was once seen as the quickest solution for relieving potentially life-threatening malignant SVC syndrome. However, it is no longer the preferred choice for most patients due to several reasons: endovascular recanalization with or without stenting is faster and more effective at relieving symptoms, especially in patients with severe symptoms; RT before biopsy can hinder obtaining an accurate histologic diagnosis; and RT can be delayed till severe symptoms are alleviated through endovascular techniques and a confirmed biopsy is obtained [7]. Since symptomatic obstruction often develops over weeks, deferring RT until a full diagnostic work-up is completed is generally safe for stable patients [7].

For patients with SCLC, non-Hodgkin lymphoma (NHL), or germ cell cancer, initial chemotherapy is preferred for non-life-threatening SVC syndrome, especially if they have not been treated before, as chemotherapy typically leads to rapid symptom relief and long-term remission [7]. Symptomatic improvement usually occurs within one to two weeks. Chemotherapy alone resolves symptoms in 77% of SCLC cases, with a 17% recurrence rate [7]. SVC syndrome does not necessarily indicate a poor prognosis, and for heavily pretreated patients, alternative therapies like RT or stent placement may be needed [5]. Therefore, deeming the patient stable enough for a safe discharge with a disposition to his home, along with specific instructions to follow up with the hematology/oncology team for initiation of chemotherapy, was a reasonable and sound plan.

## Conclusions

The case of MM leading to SVC syndrome underscores the rarity of this condition. While SVC syndrome is often associated with malignancies such as lung cancer and lymphoma, melanoma rarely presents in this manner. The patient's presentation with a mediastinal mass causing mild SVC syndrome highlights the unpredictable nature of melanoma metastasis. Despite the extensive history of melanoma management, this case illustrates how melanoma can still metastasize decades later, resulting in significant complications. The rarity of melanoma causing SVC syndrome makes each case a valuable addition to the medical literature, providing insights into the varied presentations and emphasizing the need for a tailored approach to diagnosis and management.
